# Digital mobile technology facilitates HIPAA-sensitive perioperative messaging, improves physician-patient communication, and streamlines patient care

**DOI:** 10.1186/s13037-015-0070-9

**Published:** 2015-05-23

**Authors:** Chad R. Gordon, Kameron S. Rezzadeh, Andrew Li, Andrew Vardanian, Jonathan Zelken, Jamie T. Shores, Justin M. Sacks, Andres L. Segovia, Reza Jarrahy

**Affiliations:** Division of Plastic & Reconstructive Surgery, Department of Surgery, The Johns Hopkins University, Baltimore, MD USA; Division of Plastic & Reconstructive Surgery, Department of Surgery, David Geffen School of Medicine at UCLA, 200 UCLA Medical Plaza, Suite 465, Los Angeles, CA USA; Department of Pediatrics, David Geffen School of Medicine at UCLA, Los Angeles, CA USA

## Abstract

**Background:**

Mobile device technology has revolutionized interpersonal communication, but the application of this technology to the physician-patient relationship remains limited due to concerns over patient confidentiality and the security of digital information. Nevertheless, there is a continued focus on improving communication between doctors and patients in all fields of medicine as a means of improving patient care. In this study, we implement a novel communications platform to demonstrate that instantaneously sharing perioperative information with surgical patients and members of their support networks can improve patient care and strengthen the physician-patient relationship.

**Methods:**

423 consecutive patients scheduled to undergo elective surgical procedures were offered complimentary registration to a secure, web-based service designed to distribute perioperative updates to a group of recipients designated by each patient via Short Message Service (SMS) and/or email. Messages were created by attending surgeons and delivered instantaneously through the web-based platform. In the postoperative period, patients and their designated message recipients, as well as participating healthcare providers, were asked to complete a survey designed to assess their experience with the messaging system. Survey results were statistically analyzed to determine satisfaction rates.

**Results:**

Of the qualifying 423 patients, 313 opted to enroll in the study. On average, patients selected a total of 3.5 recipients to receive perioperative updates. A total of 1,195 electronic messages were generated for distribution to designated recipients during the study period and delivered to recipients located around the world. There were no documented errors or failures in message delivery. Satisfaction surveys were completed by 190 users of the service (73 %). Respondents identified themselves as either patients (*n* = 48, 25.5 %), family/friends (*n* = 120, 63.8 %), or healthcare providers (*n* = 15, 12 %). Satisfaction with the service was high: 94.2 % of users “*enjoyed this software*” and and 94.2 % of family/friends “*felt more connected to their loved ones during surgery*.” 92.5 % would “*recommend their loved ones sign up for this service*". Ninety percent of patients who completed the survey reported “*an improved hospital experience*”

**Conclusion:**

Digital communications platforms can facilitate the immediate transfer of HIPAA-compliant data to patients and their designees. Such systems can greatly improve the level of communication between physicians, patients, and patients’ families and caregivers. All types of users, including healthcare professionals, patients, and their loved ones, recorded high levels of satisfaction. Based on these observations, we conclude that mobile digital communications platforms represent a way to harness the power of social media to enhance patient care.

## Introduction

Digital media in general, and the smart phone in particular, have revolutionized the way human beings interact with one another. Despite the impact of mobile technology on global communication and the long-held axiom that a successful physician-patient relationship should be founded on effective communication, the medical community has been slow to formally implement this technology into clinical practice [1]. There are numerous reasons why physicians may avoid the regular use of mobile devices to communicate with their patients. These may include time constraints, a desire to maintain a sense of detachment or professionalism, and concerns over possible patient privacy issues—particularly as they relate to violations of the Health Information Portability and Accountability Act (HIPAA) [2].

Even with these concerns, the impetus to incorporate digital media into the physician-patient relationship has existed for some time. For years, telemedicine platforms have offered an opportunity for healthcare providers to connect with their patients; unfortunately, this modality is associated with barriers to access including high equipment costs and the need for reliable, industrial-grade broadband internet connectivity [3]. By contrast, the ideal digital physician-patient interface would make use of a ubiquitous, low-cost device that is easy to use for both the healthcare provider and the patient.

Within the healthcare community, surgeons and their patients are uniquely poised to benefit from the integration of digital media into the patient experience. More frequent communication between surgeons, their patients, and their patients’ families would be of great benefit to all involved, especially during the perioperative period. Given the risks associated with surgical procedures and supporting a loved one through surgery, updates from the operating room can be invaluable to members of the patient’s support network. In this study, we describe our experience with a novel communications platform to demonstrate that instantaneously sharing perioperative information with surgical patients and their loved ones can enhance the physician-patient relationship.

## Materials and methods

We conducted a prospective multicenter study of patients presenting to participating institutions for elective surgical procedures over a two-year period. The study design and all study materials, including consent forms and survey instruments, were approved by the Institutional Review Board (IRB) at the Johns Hopkins University School of Medicine.

### Patient enrollment

During the study period, all patients presenting to participating centers throughout the United States for elective inpatient or outpatient surgical procedures were offered enrollment in the study. Participating facilities included academic medical centers, tertiary care referral centers, community-based hospitals, and private practice offices affiliated with outpatient surgery centers. Enrollment was not offered to any patient who required urgent or emergent surgical intervention. Surgical procedures were performed in the following disciplines: plastic and reconstructive surgery, otolaryngology, orthopedic surgery, pediatric surgery, vascular surgery, and surgical oncology.

### Electronic communications

Enrolled patients were asked to complete an online registration process with a web-based service that provided a digital platform for the creation and distribution of electronic messages related to patient care during the perioperative period. (MDconnectME, Inc., Philadelphia, PA). Once registered with the service, patients entered a list of individuals—including their email addresses and phone numbers—that they wished to designate as recipients of electronic messages related to the patients’ health care. All healthcare providers and ancillary staff who would be involved in the perioperative care of the enrolled patients and who might be involved in the process of sending electronic messages also registered with the online service. This included surgeons, nurse practitioners, physician assistants, nurses, and administrative staff. Physicians, healthcare staff, and administrative staff were all trained on how to use the communications platform until they were well versed in its proper functioning and utilization.

Patients were counseled that the service was designed to allow healthcare professionals to deliver instantaneous, secure, confidential, and HIPAA-compliant updates on their status of to a list of recipients of their designation. Surgeons or other healthcare providers participating in the patient’s care sent messages through a secure server via IM, SMS, text, or email. Messages were compatible with all popular operating systems that currently occupy the smart phone market (e.g., Mac iOS™, Blackberry™, Android™, etc.). Only recipients identified by the patient during the study enrollment and software registration processes and would have access to these electronic messages.

**Fig. 1:**
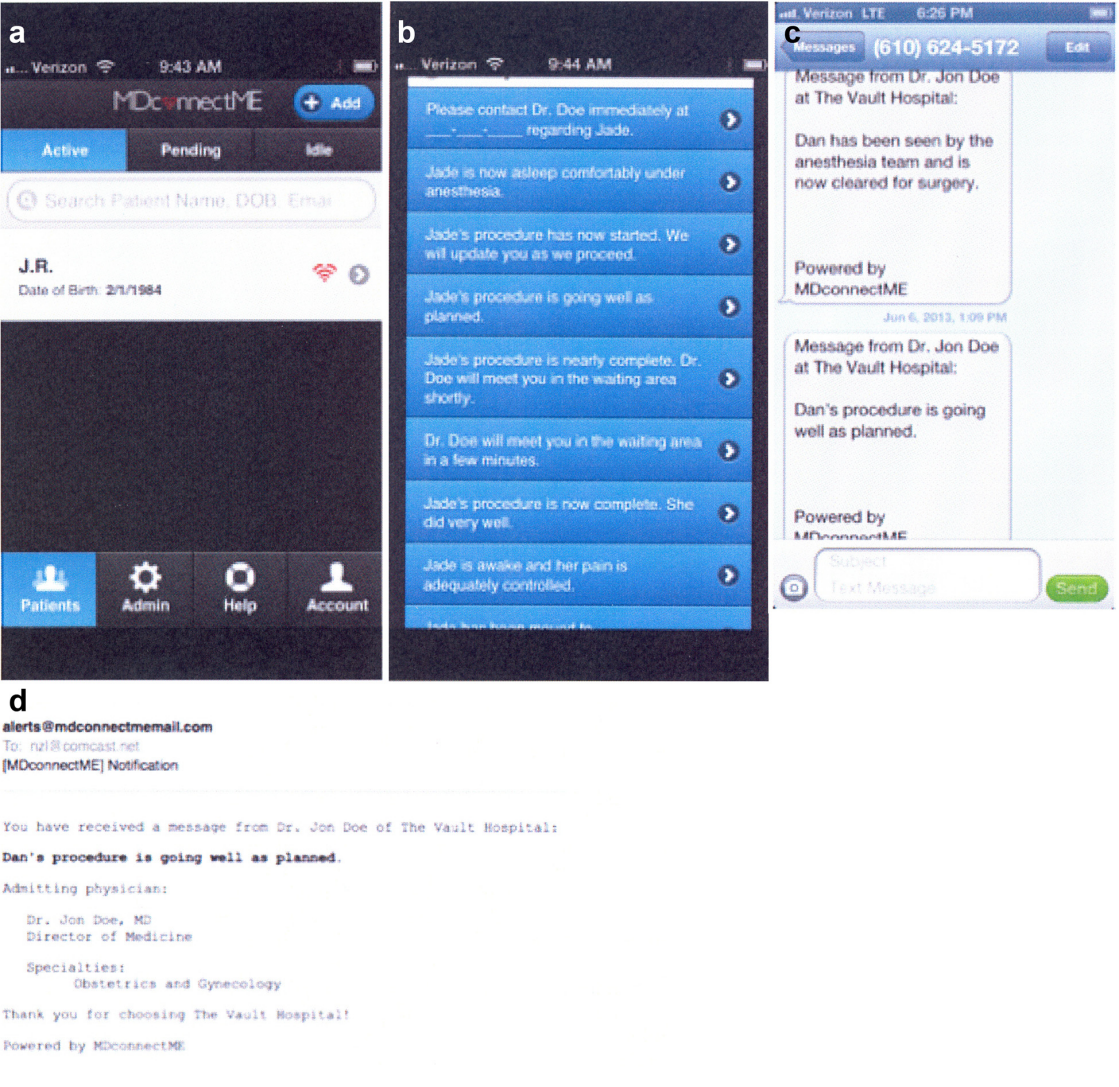
Sample screenshots depicting instantaneous perioperative messaging. These images show the simple user interface (UI) used by physicians or other healthcare professionals who sent messages to a HIPAA-compliant, patient-designated lists of recipients. Providers were able to select the patient they are treating (**a**) and choose a message from a customizable list of pre-populated message fields during surgery (**b**). In turn, recipients instantaneously received these messages on their hand-held devices, either via SMS (**c**) or email (**d**)

Finally, during the perioperative period (i.e., before, during, and immediately after an actual surgical procedure, and then during the postoperative recovery period) surgeons and other involved healthcare providers used the secure messaging system at their discretion to generate and instantaneously deliver relevant electronic messages to each patient’s distribution list (Fig. 1).

### Evaluation and analysis

**Fig. 2:**
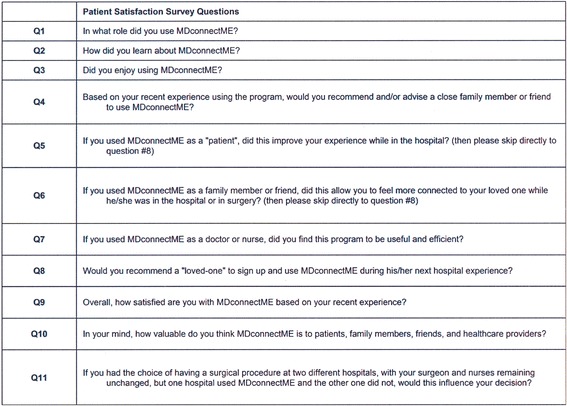
Survey instrument used to assess satisfaction with the perioperative digital messaging service

Within forty-eight hours of completion of the procedure (for outpatient surgeries) or after discharge (for inpatient procedures) the patient, their designated message recipients, and all healthcare and administrative personnel associated with the patient were asked to complete an anonymous, web-based, electronic survey aimed at assessing each user’s experience with the electronic messaging service. (Fig. 2) Survey results were complied in aggregate and subject to statistical analysis.

## Results

During the study period, 423 consecutive qualifying patients were offered enrollment in the study. Of these, 313 opted to enroll and successfully completed the online registration process. A total of 1,609 users, including patients, designated message recipients, and healthcare providers, registered for the study.

On average, patients designated 3.5 individuals to receive perioperative electronic messaging related to their healthcare delivery. Patients listed an average of one emergency contact. Physicians and other healthcare providers drafted a total of 1,195 electronic messages for distribution to recipients during the study period. Many recipients were contacted through multiple means based on preferences listed during the registration process (e.g., message delivery via SMS *and* email). A total of 10,634 electronic messages were successfully delivered to recipients. Based on Internet Protocol (IP) address tracking, messages were noted to be delivered to destinations in several countries around the world. There were no errors or failures in message delivery reported by patients or their designated recipients, healthcare personnel, administrative staff, or support staff, indicating a 100 % successful transmission rate. Of the total messages sent, 5,047 were delivered via email (47 %) and 5,587 were delivered via SMS (53 %).

**Fig. 3:**
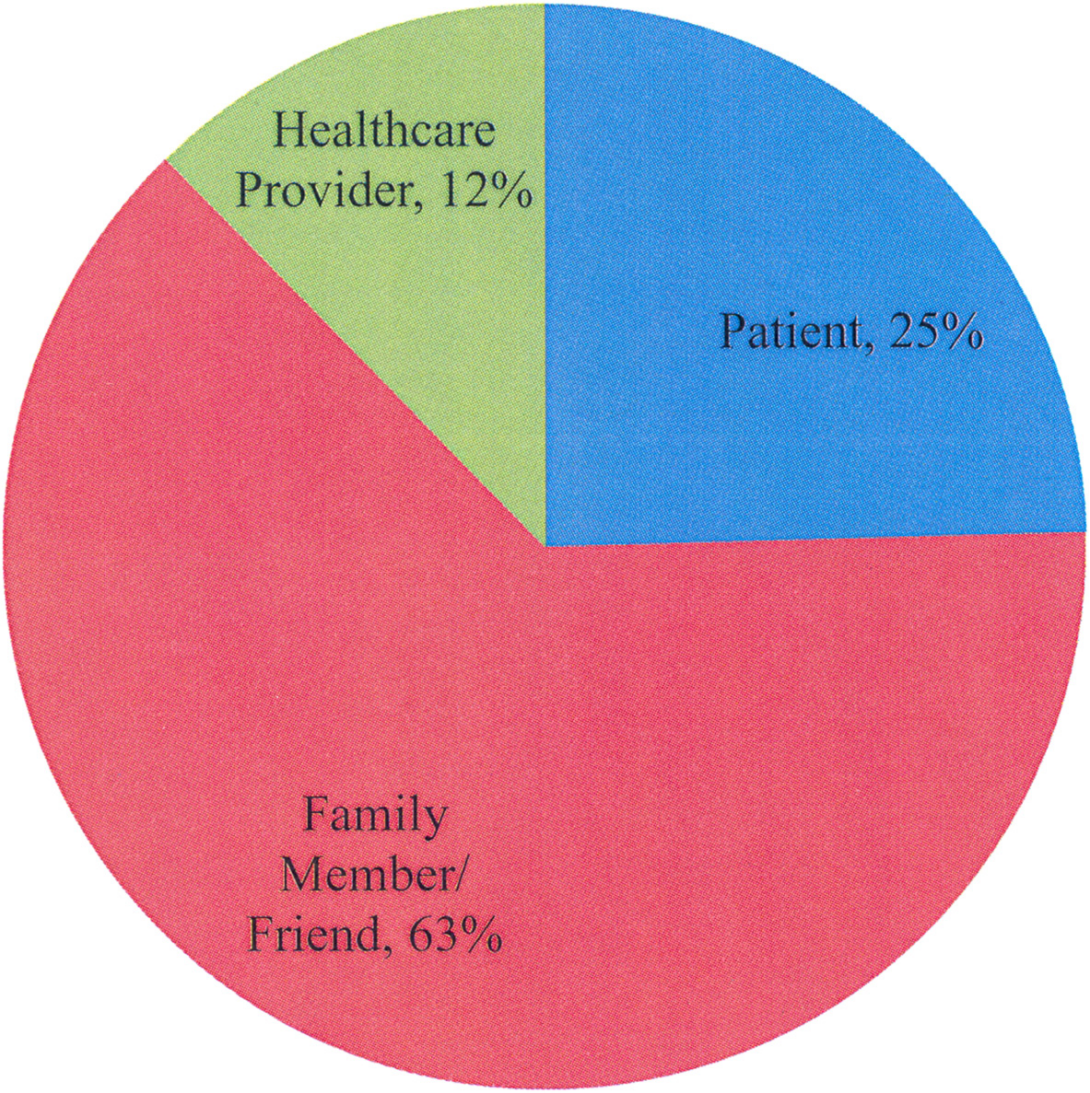
Satisfaction survey respondents by service user type

**Fig. 4:**
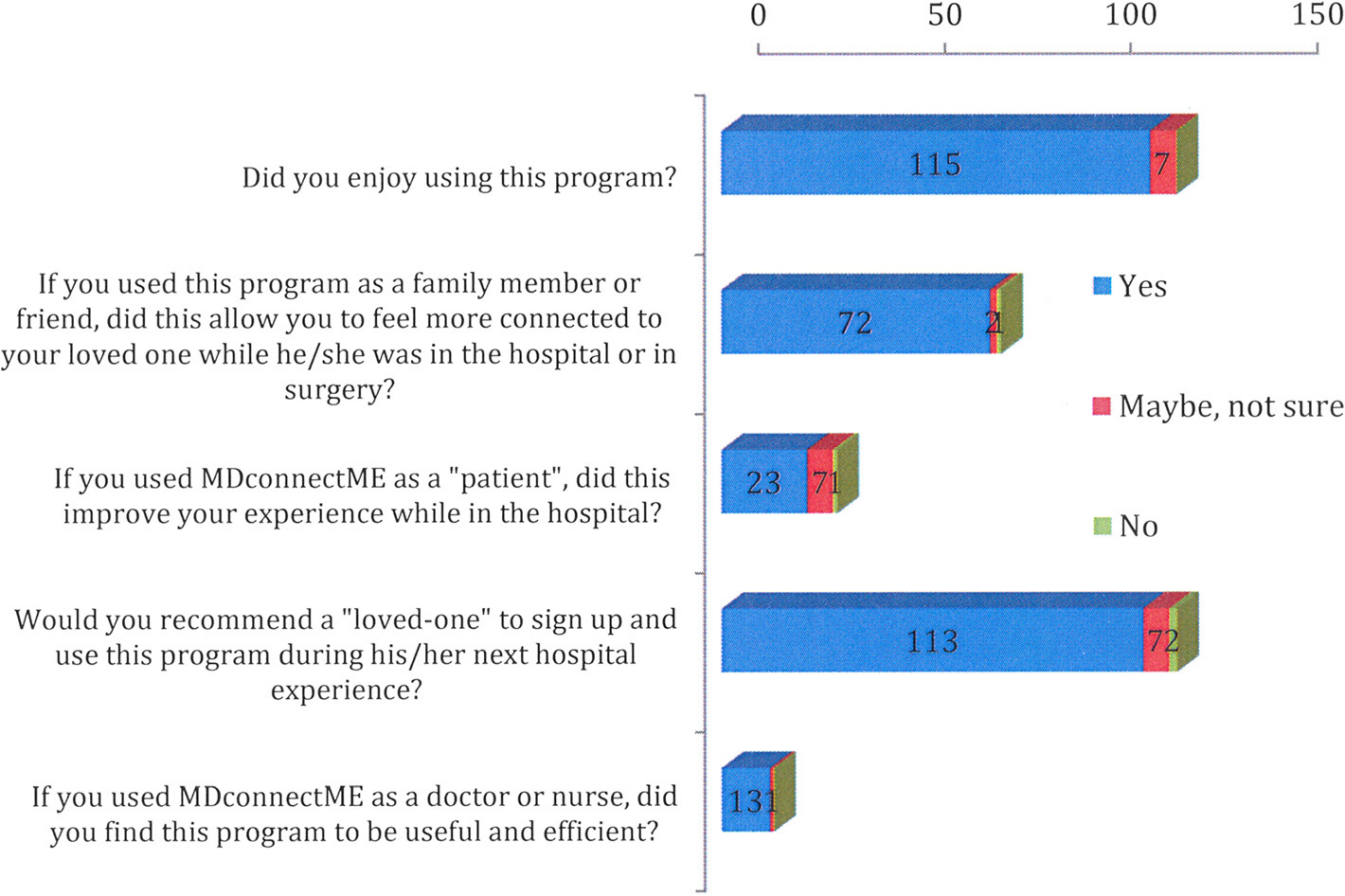
Responses to select questions from the user satisfaction survey. Numerical values represent the number of respondents per response

803 online feedback surveys were distributed to study participants. One hundred ninety of these surveys were completed, yielding a survery response rate of 24 %. Of the respondents, 53 identified themselves as patients (28 %), 120 as family and friends (63 %), and 15 as healthcare workers (8 %) (Fig. 3). The majority of respondents were satisfied with their experience using the messaging service; 94.3 % of users replied in the survey that they “*enjoyed this software*” and 92.6 % replied that they would “*recommend to their loved ones to sign up for this service.*" Most patients (74.2 %) who completed the survey reported “*an improved hospital experience,*” and 96 % of responding family members and friends “*felt more connected to their loved ones during surgery*” (Fig. 4). One-half of all patients surveyed claimed they would choose a hospital based on the availability of a perioperative communications tool, if all other factors remained equal. Of note, family members offered several useful queries and suggestions regarding this software. The most common questions pertained to frequency of messaging: patients and their families were interested in either receiving fewer or more frequent messaging from healthcare professionals. Patients’ families were also interested in receiving texts at regular intervals pertaining to their loved one’s status during the post-operative period (i.e., during the entire hospital course).

## Discussion

The number of digital mobile applications (“apps”) focused on medicine and healthcare has increased drastically throughout the past decade, with greater than 40,000 apps currently available [4, 5]. Despite this impressive volume of software, a reliable mobile communications platform designed specifically for use by physicians, their patients, and their patients’ support networks is not currently available. Our study demonstrates that there is a potential market for such a product: we have shown that smart phone-based communication between healthcare providers, their patients, and their patients’ designees, greatly improves satisfaction with the perioperative experience. Our results indicate that the use of this technology significantly enhances connectivity between surgical patients and those they identify as their closest advocates. The vast majority of those surveyed stated that they would recommend this type of product to their loved ones. The results of this study indicate that an accessible digital communications platform capable of facilitating physician-patient communication would be well received in today’s medical marketplace.

In the perioperative surgical setting, which this study has focused on, the current methods that are used to communicate with patients’ relatives are highly variable and often antiquated. At many institutions, circulating nurses or other operating room staff are called upon by the operating surgeon to deliver status updates via telephone to family members sitting in a remote waiting room. Such a phone call is typically brief and usually provides only limited information (e.g., “all is going well”). While this method of communication can be meaningful, it has significant disadvantages. First, the surgical waiting areas of many hospitals often lack privacy and the opportunity for discretion. Family members might be uncomfortable receiving sensitive information regarding a potentially stressful situation in a public setting. Private mobile messaging liberates families from the waiting room, allowing them to find a peaceful environment of their choice where they can await news. Next, when telephone updates are delivered to a singular family member, that person might then have the unwelcome burden of transmitting sensitive information to a host of additional members of the support group. This might add additional stressors to the family member who takes the call. By allowing messages to be delivered to an unlimited number of individual recipients, mobile messaging spares any one member of the larger support group from serving as a secondary messenger. This would be particularly useful in facilitating updates for friends or family members that are not physically present at the hospital at the time of surgery, including relatives who might live out of state but whom the patient would like to keep informed. In addition, by allowing surgeons to generate an unlimited number of custom templates, messages of any level of detail can be drafted and delivered. Recipients would therefore be able to receive messages with greater meaning and relevance regarding their loved one’s status. This flexibility also alleviates the ancillary staff who are making the update phone calls of the burden of trying to provide a higher level of information than the standard curt summary that is usually provided, which is often followed by more probing questions from the family member on the receiving side. It further frees all operating room staff, including the surgical team, to focus on the task at hand: completing the operation safely without any lapses in focus or concentration.

While this study focused on the discipline of surgery, we can easily imagine the benefits of this type of communications application outside of the surgical model that we have studied. The results of any laboratory, pathology, or radiography studies can be instantaneously shared with concerned family members all over the globe. In the critical care setting, doctors can communicate with a patient’s extended support group more efficiently and in a less stress-inducing environment than the typical crowded consultation room outside of the intensive care unit. News of the arrival of a newborn baby boy or girl can be sent to eager aunts, uncles, and grandparents back home. The opportunities for enhancing communication pertaining to medical issues are seemingly limitless.

Effective communication between physicians and patients lays the foundation for an optimal patient experience with high satisfaction. Patients who feel they have more effective lines of communication with their physicians tend to be more compliant, more successful in modifying unhealthy habits, and engage more actively in the management of their own health [6, 7]. Improving communication during perioperative period may have downstream effects, including a decline in adverse outcomes. This may be particularly relevant when members of a patient’s support network will actively be providing care to the patient throughout the duration of their surgical or medical illness, and therefore would benefit from the most current information related to their loved one. Furthermore, studies have shown that failures in communication alter the surgeon-patient relationship and can lead to poor outcomes, ultimately increasing the chances of malpractice suit [8, 9]. Occasional lapses in communication have also been found to decrease word-of-mouth recommendations by patients to their friends and family [10].

A recent study of 78 medical practices utilizing electronic methods to communicate with their patients concluded that the use of digital media in the physician-patient relationship improves access to care, saves patients time, and increases overall patient satisfaction [11]. Physicians reported that electronic communications systems improved overall productivity, as sending patients secure digital messages was less time consuming than attempting to reach them by phone. Yet another study has examined the impact of implementing a communications training program for physicians and other healthcare providers caring for cardiothoracic surgery patients. The authors found that those patients cared for by healthcare workers with communications training experienced fewer post-operative complications [12]. While the sample sizes of these types of studies are limited, the relationship between improved provider-patient communication and better patient care is intuitive and worthy of future study.

In summary, our results illustrate the benefit of formally implementing mobile digital communications technology into the doctor-patient relationship. It can be done simply, with minimal cost, and can have far-reaching effects in streamlining patient care, improving patient satisfaction with that care, and improving medical and surgical outcomes. While we acknowledge study weaknesses including a low survey response rate, we believe meaningful conclusions can be drawn from our findings. We believe further study of the feasibility of incorporating communications platforms such as the one used in this study at the enterprise level are warranted.

## Conclusions

We have shown that a digital communications platform can facilitate the transfer of HIPAA-compliant data to patients and their friends and family. We have demonstrated this in a cohort of surgical patients, but our observations can be extrapolated to any medical specialty. Such systems have the potential to improve the level of communication between physicians, patients, and patients’ loved ones. We have observed high levels of satisfaction using such a system from healthcare providers, patients, and their designees. Higher levels of satisfaction and greater inclusion of a patient’s support network may translate into improved outcomes, and is worthy of continuing study.
